# Computed tomography head and facial bones review of a 2700 year old Egyptian mummy

**DOI:** 10.1259/bjrcr.20190076

**Published:** 2020-09-29

**Authors:** Paul Lockwood, James Elliott, Andrew Nelson, Samantha Harris

**Affiliations:** 1 School of Allied and Public Health Professions, Faculty of Health and Wellbeing, Canterbury Christ Church University, Kent, UK; 2 Radiology Department, Maidstone and Tunbridge Wells NHS Trust, Kent, UK; 3 Departments of Anthropology and Chemistry, Western University, Ontario, Canada; 4 Maidstone Museums, Kent, UK

## Abstract

CT scanning techniques used in head and facial bones examination in the clinical environment can also be transferable to the imaging of post-mortem cases as a novel non-destructive and non-invasive investigation in forensic cases. We describe a study of the head and facial bones of a 2700-year-old Egyptian mummy. Cross-sectional investigation can lead to discovering unknown information of skeletal and soft tissue structures and anatomy to contribute to the knowledge of preserved mummified remains and the practice of palaeoradiology.

## Case presentation

The Egyptian mummy ‘Ta Kush’ known as ‘The Lady of the House, Daughter of Osiris’ (god of the afterlife) from the 25th Egyptian Dynasty (third intermediate period of Egypt is known as the Nubian Kushite Empire), was brought to England from Thebes (Egypt) in the 1820s and was first examined by the British Museum in 1843, before being donated to Maidstone Museum (Kent, UK). Inscriptions on the Inner wooden coffin described her name, marriage status, her origin of Northern Sudan, and burial site in Thebes (Egypt)^1^ ca 700–650 BC.

## Investigation

**Figure 1.**
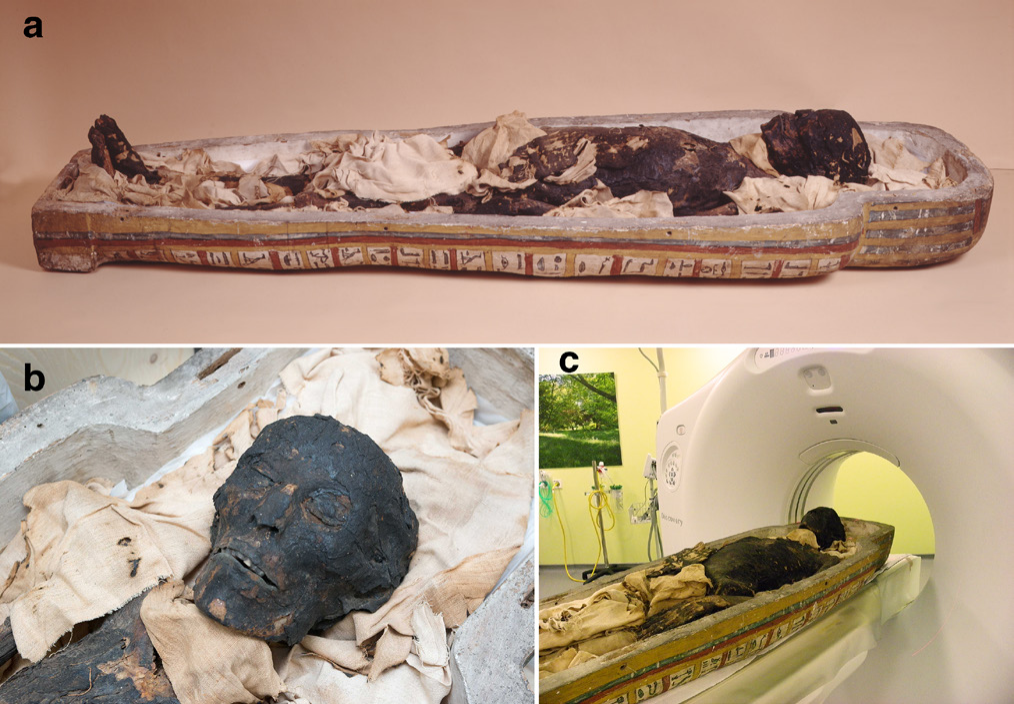
(a) Ta Kush in the inner wooden coffin. (b) Ta Kush uncovered face. (c) Ta Kush in the CT scanner.

In July 2016, Maidstone Museum (Kent, UK) was awarded a Heritage Lottery Fund grant for the modernisation of its Egyptology Gallery, which included the commissioning of a local hospital to CT image ‘Ta Kush’ (November 2016). Standard CT head protocols were applied (140 kv, 260 mA),^2,3^ exposure factors did not apply tube current modulation (automatic exposure control) optimisation that typically limits radiation in living tissue examinations. Contiguous axial slices (0.625 mm) were acquired from the vertex to mid-seventh cervical vertebra on a GE 750HD Discovery (General Electric Healthcare Technologies, Waukesha, WI) CT scanner. Reformats were generated and reviewed in axial, coronal and sagittal (Medixant, RadiAnt DICOM Viewer v. 4.6.9, Poznan, Poland). Due to the fragility of the cadaver specimen, it was recommended the mummy remained wrapped in its original fabric laying and supported on the inner wooden anthropoid coffin to reduce displacement or damage to the remains (Figure 1a–c). The wooden coffin being of a dry and porous consistency with a degree of air trapped within the material (organic in nature) was a low attenuating material^4^ providing a Hounsfield unit (HU) of −500 to −600 HU and was not clearly visualised on cranial soft tissue narrow windows, general soft tissue windows or bone windows. The wooden surfaces of the coffin were only visualised on wide lung windows and provided no imaging artefacts.

## Findings

**Figure 2.**
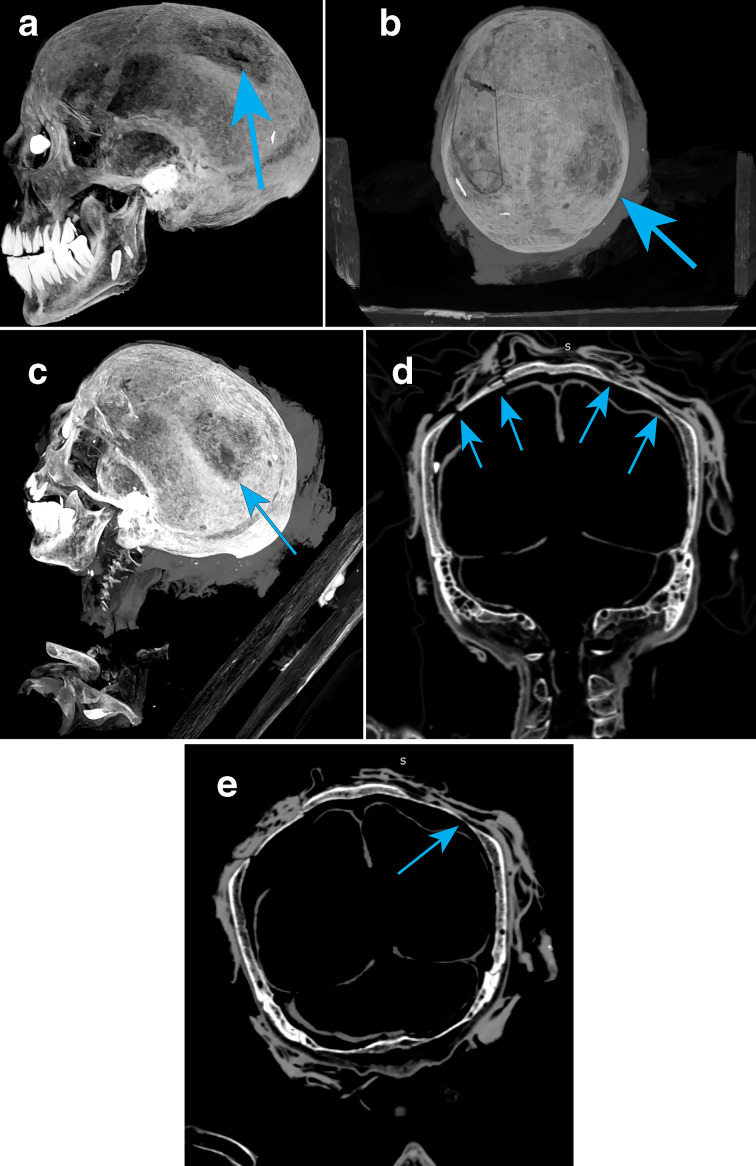
(a-e) Parietal bone thinning.

**Figure 3.**
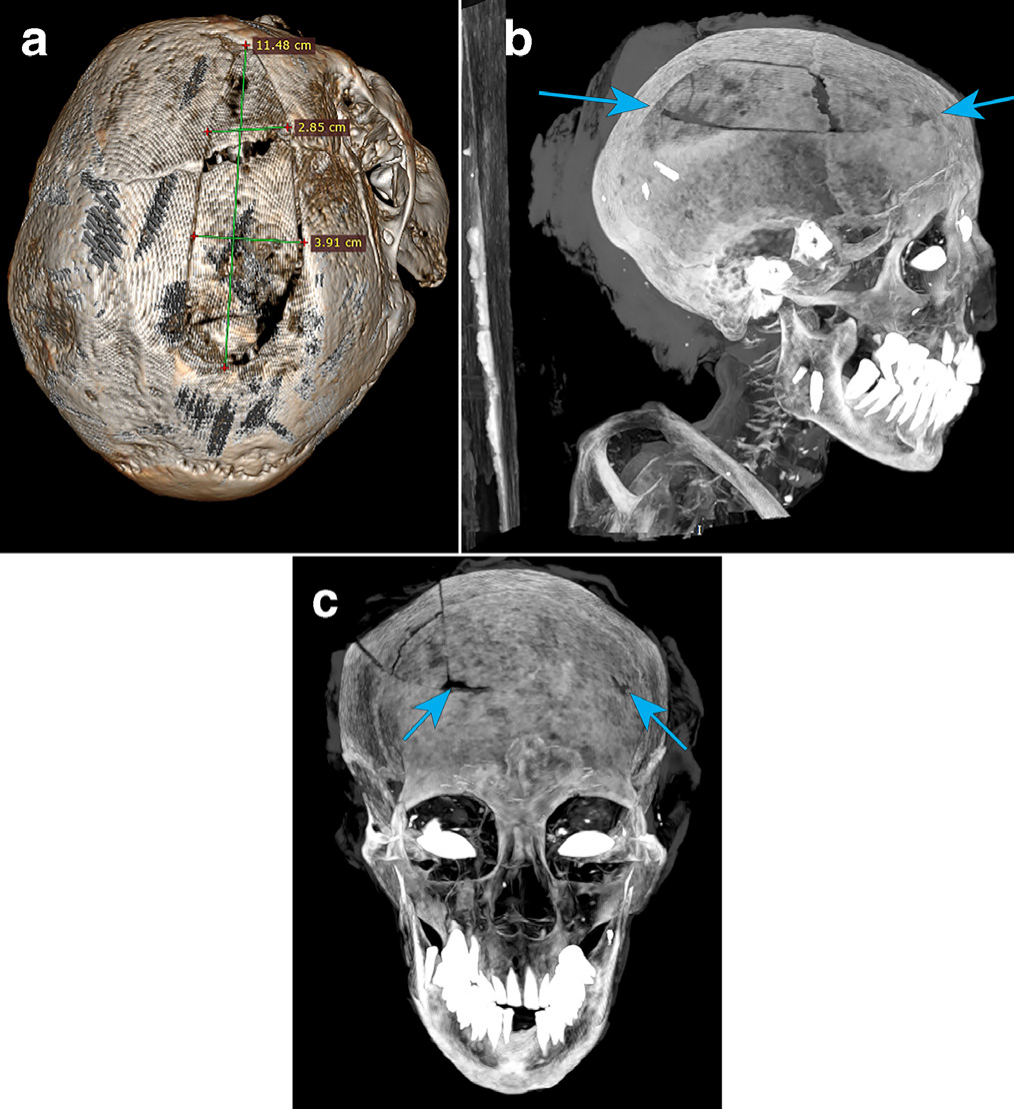
(a-c) Right parietal bone fracture.

Examination of the cranium identified localised thinning of the left posterior parietal bone, with an ovoid central area of parietal thinning (1.2 x 1.6 cm; Figures 2a–e and 3e), the cortex of the parietal bone was intact but with a reduced trabecular bone (Figure 2a–e). Additional multiple smaller lytic areas surrounding the central depression (5.1 × 5.2 cm) that penetrated the bone cortex and diploe were evident.

Similar parietal thinning on the right side gave the vertex a squared impression (comparable to a mesocephalic head) with reduced internal parietal diploe of the cancellous bone (Figure 2d–e).

A smaller but prominent occipital outpouching bone feature was noted posteriorly giving the occipital bone an appearance of hair in a bun shape (not related to the external occipital protuberance) of normal variant anatomy.

A right parietal fracture extending through the right coronal suture to the right frontal bone (5.4 cm in diameter), extending inferiorly across the right frontal bone (2.8 cm) and inferiorly across the parietal bone (3.9 cm), then extending along the bottom of the parietal bone (6 cm) displayed a rectangular shaped craniotomy (Figure 3a–c). The area of bone across the coronal suture appears widened and jagged. Towards the posterior of the rectangular fragment, there was a collection of small fragments, possibly an incision point, with a similar frontal incision point in the left frontal bone (8.82 x 7.35 mm). These may be from the documented 1846 Victorian importation customs search, or the autopsy reported by Diamond (1846).^1^

The occipital bone was intact as were the left and right temporal bones, there were some pacchionian impressions (arachnoid granulations) within the trabecular pattern, but within normal limits and not extending to the surface cortex. It was noted that no internal hyperostosis frontalis was evidenced from a menopausal metabolic change to the inner table of the frontal bone.

**Figure 4.**
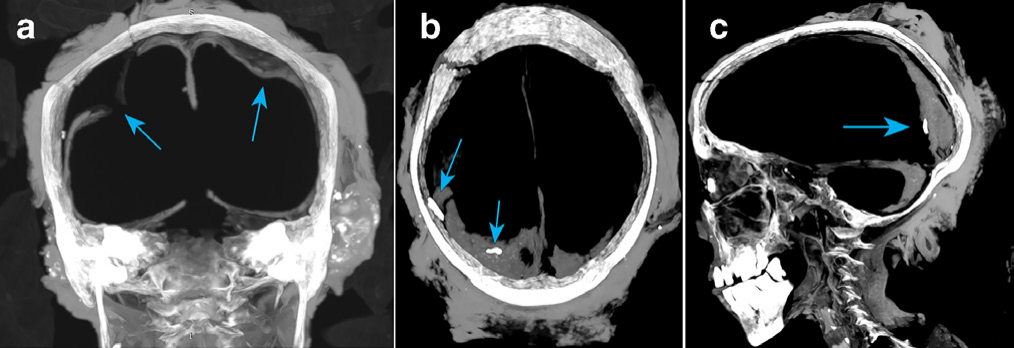
(a-c) Cerebral and cerebellar dura and tentorium.

The cerebral and cerebellar dura was still intact, albeit no longer attached to the inner table of the cranium, but connected to the tentorium. The right dura appeared torn underneath the right parietal fracture, which extended through the medial and lateral walls of the right tentorium and into the right cerebellum floor (Figure 4a). Two hyperdense artefacts (1500–1900 HU) were identified, one between the cranium and the right lateral dura, and one on top of the posterior skull vault contents, possibly from the overlying fracture or the implement causing the fracture (Figure 4b–c).

The thin layer (9 mm) of dehydrated and dissected contents of the cerebral hemispheres at the posterior of the cerebrum and cerebellum was assumed to be the brain, displaying a heterogeneous pattern with a density of 40 HU interspaced with mixed aerated content (−75 HU).

The internal and external acoustic meatus, the carotid canal and tympanic cavity were visible and undisturbed. The mastoids appeared unremarkable and aerated; it was observed that the left sigmoid sinus was larger than the right (normal variant).

**Figure 5.**
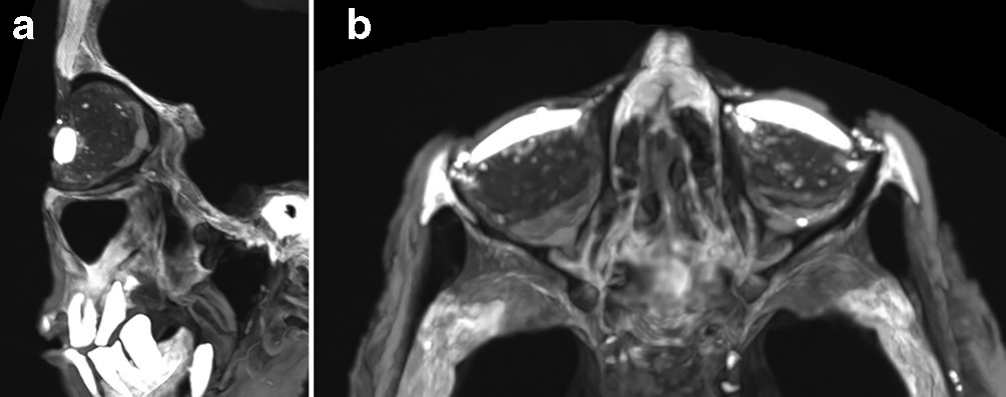
(a-b) Artificial lens with orbital packing.

Upon examination of the facial bones and paranasal sinus, bilateral orbital artefacts (high-density scleral shell) were identified over the eye of ovoid shape (2.46 cm in length, 1.2 cm in diameter, 2600 HU density), assuming an appearance of an artificial eye lens. Underlying orbital packing material of mixed −450 to −700 HU was seen (Figure 5a–b). The original globe and vitreous body of the eye would have dehydrated; it was unknown if the orbital remains have been injected with a material to maintain their shape and structure, as on the axial slices the extraocular muscles of the medial and lateral rectus are visible. The orbits have the appearance of a ruptured globe with air pockets within or have undergone enucleation/evisceration and replacement with packing. Remnants of the optic nerve of the right and left eye attached to the posterior part of the globe are intact.

**Figure 6.**
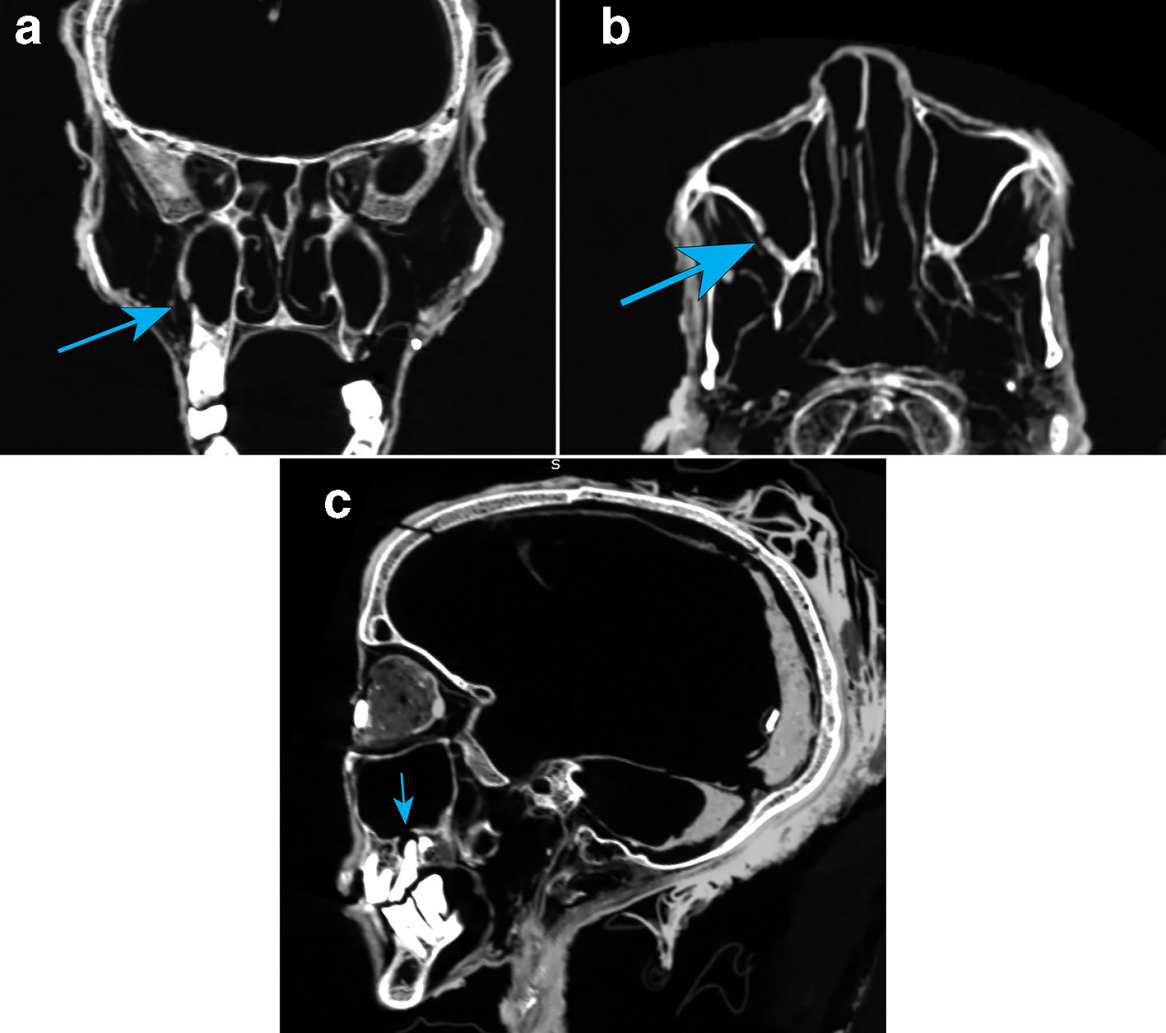
(a-b) Fracture of the right lateral maxillary lower wall. (c) Upper second right molar root extending into maxillary sinus.

The visualised paranasal sinuses were well-aerated. The maxillary, ethmoid, sphenoid and frontal sinus demonstrated no obvious obstruction or wall thinning, scalloping, erosion or fistula from chronic sinus disease, with normal ventilation and sinus drainage seen in the outflow tracts. The right sphenoid was more substantial than left (normal variant), the left frontal sinus larger than the right (normal variant), the left nasal conchae larger than the right (normal variant of ethmoid bulla). Normal bony septa, normal cribriform plate and fovea ethmoidalis, straight nasal septum and remnants of the middle and inferior turbinate’s, and patent lamina papyracea were all clearly identifiable. There was no evidence of a post-mortem surgical incision route (transnasal craniotomy) into the cranial vault as commonly observed with excerebration procedures for embalming, as noted cerebral material *in situ*, and the cribriform plate was intact. A fracture was noted through the right lateral maxillary lower wall, which tracks inferiorly and is closely associated with the abscess at the tip of the root of the right upper molar. The zygomatic arches were intact, as were the nasion and inferior and superior orbital margins (Figure 6a–c).

**Figure 7.**
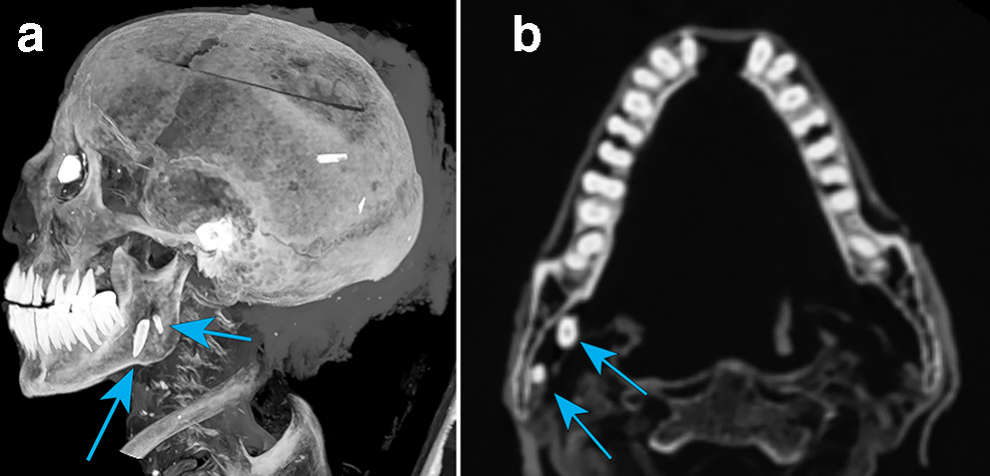
(a-b) Tooth artefacts beside right mandible.

The upper aerodigestive tract appeared normal, as did the nasopharynx, oropharynx, pharynx and hard palate, which was intact and empty. Skin surface under the chin, the inferior surface of the mandible appears to have an open wound extending into the oropharynx with the soft tissue tongue missing. The mandible and bilateral temporomandibular joints appeared normal. Review of the dental anatomy within the mouth indicated erupted lower third molars (wisdom teeth). The teeth present had a considerably worn down outer enamel coating, with the crowns missing and anterior angulation of the lower third molar tooth. The lower mandibular teeth of the left incisor and both central incisors were absent with visible sockets. The two missing lower central incisors are possibly the two artefacts resembling tooth shapes seen inside the right angle of the mandible (gonial angle) and within the oropharynx, and probably disrupted post-mortem (Figure 7a–b). The upper maxilla teeth noted an absence of the second and third molars on the left and a missing third molar on the right. The upper second molar on the right displayed a root extending into the maxillary sinus (Figure 6c) with surrounding bony erosion possibly related to abscess formation. Likewise, both frontal and central incisor roots protrude out through the anterior surface of the maxilla.

## Discussion

We cannot explicitly identify a cause and effect relationship of the pathologies found in this examination of the mummy, factors at the time of death have not been recorded. They are possibly multifactorial in origin including diet, general health and comorbidities. There is the potential for HU drift due to CT manufacturer variation and post-mortem diagenesis of dry bone. Therefore, causal inference and generalisability can be challenging to determine circumstances or infer mechanisms of injuries (pre- or post-mortem occurrence). Imaging evidence of callus formation or osteoblastic bone remodelling of fractures can be helpful, but a detailed physical inspection will provide further evidence and information.

The rectangular fracture to the right parietal bone shows no osteogenic reaction or evidence of callus formation of fibrous bone growth and union, indicating the insult was perimortem or later (post-mortem damage) and not a cause of death or impact upon her health. The injury itself does not follow a typical blunt force trauma impact to fresh bone, where one would expect radiating or concentric fracture lines. Rather, it appears consistent with a sharp force trauma created with a tool point or bevelled edge. Additionally, there appear artefacts inside the cranium, which may be related to the injury sitting upon the shrunken soft tissue.

The adipocere^5^ cranial contents were similar to those found in the Padihershef’s skull,^6^ which would confirm no transnasal craniotomy into the cranial vault as evidenced by the intact ethmoid, which is often fractured when Egyptian excerebration procedures for embalming occur.^7,8^ The artificial eye lens of high-density material is similar to stone artefacts recorded in other Egyptian mummies,^9^ and is consistent with mummification practice in the 25th Dynasty.^10–12^

The bilateral parietal thinning noted around the parietal eminence (typically where ossification commences in the eighth week of life) causing the vertex to give a squared appearance provided no evidence of craniostenosis (or synostosis ‘beaten copper’ calvarial bone pattern). Examples of the vertex appearance have previously been reported with an overall frequency of 4.9% as a non-metric trait with a genetic component,^13^ an example of which was noted in the CT examination of the female Egyptian mummy Renpit-Nefert. The bone thinning in ‘Ta Kush’ was the opposite of porotic hyperostosis thickening (often found in similar areas) and representative of pacchionian depressions (or multiple arachnoid granulations), generally associated with parietal convolutions and grooves adjacent to the anterior division of the middle meningeal vessels before the sagittal sulcus of the sinus. Small defects in the superior–posterior parietal bones on either side, such as these are rare in the general population of today. The central lytic ovoid area could have been an enlarged parietal foramina anomaly with the parietal impressions involving the eminences from a venous lacuna and diploic veins and outer table of the skull where an emissary vein may have passed through the tiny holes.

Differentials to a diagnosis would encompass a metabolic cause leading to osteomalacia^14^ (inadequate bone mineralisation from either poor nutrition, endocrine factors of the parathyroid, or hormone imbalance), although hypocalcified bone tissue would result in decreased outer compact bone density not identified here. Future review of the long bones for abnormal curvature associated with osteomalacia (and associated Harris’s Lines) or osteoporosis may be helpful.

In this case study, it appears to be the compact outer surface of the parietal bone that was thinned to the cancellous diploe, and the inner table of the parietal displays a regular pattern (for dry bone). A comparable appearance is described in the CT scans of the Pisa 1 Filippo Civinini Egyptian mummy’s skull,^8^ which were attributed to a hypothesised (no evidence of) adjacent meningioma. In our case study example, there are no inner table changes to the bony architecture to support resorption due to the action of local osteoclasts stimulated by the presence of a primary tumour.

## Conclusion

Forensic imaging of archaeological human remains with advanced post-mortem change is challenging, not only due to soft tissue decomposition but also due to post-mortem mummification practices. Imaging can provide a wealth of information on patient health status, age and co-morbidities at time of death, possible causes of death, biometric data, and information on ancient Nubian Dynasty (Kushite Empire) Egyptian funeral customs.

## Learning points

1. The objective of presenting this case study is to provide within reasonable limits a comprehensive survey of a mummified CT head scan. Archaeology is becoming increasingly complex with multiple sub specialities involved (palaeoradiology, bioarchaeology, osteoarchaeology, anthropology etc.); therefore, it is difficult for one professional to be equally expert in all areas and collaboration between archaeology and clinical radiology will be a valuable exercise.
2. The case presented illustrates the difficulty in post-mortem image reporting to assign necessarily a clinical diagnosis on causes of cranial bone thinning, but a detailed report of imaging and differentials is possible.
3. Fracture ageing is a complicated process, but evidence of expected healthy metabolic bone growth (callus formation) or lack of, allows classification of this insult to the post-mortem interval.
4. Little is written about the properties of dehydrated cerebral and dural tissue to definitively provide information, due in part to the scarcity of material remains.
